# Astaxanthin Protects Retinal Photoreceptor Cells against High Glucose-Induced Oxidative Stress by Induction of Antioxidant Enzymes via the PI3K/Akt/Nrf2 Pathway

**DOI:** 10.3390/antiox9080729

**Published:** 2020-08-10

**Authors:** Tso-Ting Lai, Chung-May Yang, Chang-Hao Yang

**Affiliations:** 1 Department of Ophthalmology, National Taiwan University Hospital, Taipei 100, Taiwan; b91401005@ntu.edu.tw (T.-T.L.); chungmay@ntu.edu.tw (C.-M.Y.); 2 Graduate Institute of Clinical Medicine, College of Medicine, National Taiwan University, Taipei 100, Taiwan; 3 Department of Ophthalmology, College of Medicine, National Taiwan University, Taipei 100, Taiwan

**Keywords:** diabetic retinopathy, hyperglycemia, oxidative stress, astaxanthin, carotenoid, reactive oxygen species, apoptosis, photoreceptor cells, PI3K, Nrf2

## Abstract

Diabetic retinopathy (DR) is a major microvascular complication that can lead to severe visual impairment in patients with diabetes. The elevated oxidative stress and increased reactive oxygen species (ROS) production induced by hyperglycemia have been reported to play an important role in the complex pathogenesis of DR. Astaxanthin (AST), a natural carotenoid derivative, has been recently recognized as a strong free radical scavenger and might, therefore, be beneficial in different diseases, including DR. In this study, we evaluated the potential role of AST as an antioxidative and antiapoptotic agent in protecting retinal cells and also investigated the involvement of the PI3K/Akt/Nrf2 pathway in AST-mediated effects. We treated high glucose-cultured mouse photoreceptor cells (661W) with different concentrations of AST and analyzed ROS production and cell apoptosis in the different regimens. Moreover, we also analyzed the expression of PI3K, Akt, Nrf2, and Phase II enzymes after AST treatment. Our results showed that AST dose-dependently reduced ROS production and attenuated 661W cell apoptosis in a high glucose environment. Importantly, its protective effect was abolished by treatment with PI3K or Nrf2 inhibitors, indicating the involvement of the PI3K/Akt/Nrf2 pathway. These results suggest AST as a nutritional supplement that could benefit patients with DR.

## 1. Introduction

Diabetes is a major metabolic disease that can affect different organs through microvascular and macrovascular damages [1]. Diabetic retinopathy (DR), a leading microvascular complication of diabetes, is characterized by a progressive increase in vascular permeability, retinal ischemia and edema, and neovascularization, which can result in visual impairment and even legal blindness [2]. Despite the improved understanding of its pathogenesis and the advances in available treatments, the long-term outcome of DR remains poor owing to its complex pathogenesis [3,4]. Therefore, the continuous search for new modalities to prevent and treat this debilitating complication is essential.

Hyperglycemia induces oxidative stress and generates reactive oxygen species (ROS) within the retina [5,6]; however, the activity of cellular antioxidant enzymes responding to ROS is insufficient to prevent the consequent damages. Yeh et al. reported a positive correlation between ROS levels in vitreous fluid and the severity of DR [7]. In addition, several studies have indicated chronic oxidative stress as one of the primary causes of DR [8,9,10,11,12]. Normally, the retina already presents substantial lipid oxidation and ROS production due to the high content of unsaturated fatty acids and high oxygen uptake, which render it more vulnerable to oxidative stress damage than other tissues [13,14]. Furthermore, ROS accumulation alters the homeostasis of retinal cells, triggers cellular apoptosis, and leads to increased vascular permeability and basement membrane leakage in the retina. These pathological changes may lead to a breakdown of the blood–retinal barrier and the development of DR [15,16]. Thus, reducing oxidative stress could inhibit apoptosis in retinal cells and reduce the risk of DR progression.

Astaxanthin (AST) is a member of the xanthophyll family of oxygenated carotenoid derivatives. AST has been recently recognized as a strong free radical scavenger and an excellent anti-inflammatory agent that suppresses the expression of proinflammatory chemokines and cytokines [17,18]. The unique molecular structure of AST, which contains hydroxyl and keto moieties on each ionone ring, explains its high antioxidant activity [19]. Mechanistically, AST terminates the free radical chain reaction by donating electrons and reacting with free radicals to convert them to more stable products [19,20]. AST can, therefore, act as a powerful antioxidant in numerous organisms. In fact, AST has been attributed as having the potential to defend organisms against a broad range of diseases, including cardiovascular disease [21], ischemic brain damage [22], cataracts [23], diabetes [24], and diabetic nephropathy [25,26]. AST has also shown protective effects in ocular diseases, including neuroprotection in retinal ganglion cells [18], suppression of choroid neovascularization development [27], and reduction of endotoxin-induced uveitis [28].

Recent studies have shown that AST can activate the Nrf2–antioxidant responsive element (ARE) pathway in different cell types and organs [29,30,31,32,33]. The Nrf2–ARE pathway is an important endogenous mechanism that can attenuate oxidative stress within the cell [34,35]. Nrf2 induces the expression of Phase II enzymes, including NAD(P)H dehydrogenase (NQO1) and heme oxygenase-1(HO-1), thereby limiting the subsequent generation of ROS and promoting the formation of antioxidant bilirubin [36,37,38,39]. An earlier study indicated that retinal pigment epithelial (RPE) cells could be protected against oxidative damage by activating the Nrf2–ARE pathway and increasing expression of Phase II enzymes [40]. A later study further showed the crucial role of the PI3K/Akt pathway in modulating Nrf2–ARE-dependent protection against oxidative stress in RPE cells [41].

Therefore, in this study, we hypothesized that AST could counteract high glucose-induced oxidative stress and attenuate oxidative stress-induced apoptosis by modulating Nrf2 expression in retinal photoreceptor cells. To test our hypotheses, we first investigated the role of high glucose-induced oxidative stress in initiating retinal photoreceptor cell (661W) apoptosis and evaluated the antioxidative and antiapoptotic effects of AST. Moreover, we analyzed the modulatory effect of AST on Nrf2 expression and analyzed the related signaling pathway. Our results showed that AST reduced high glucose-induced oxidative stress and attenuated apoptosis of photoreceptor cells by induction of antioxidant enzymes via the PI3K/Akt/Nrf2 pathway.

## 2. Materials and Methods

### 2.1. Cell Culture and Experimental Design

In this study, we used the 661W cell line, obtained from Dr. M. Al-Ubaidi (University of Houston), to evaluate the response of photoreceptor cells to AST treatment. The 661W cells, exhibiting biochemical features characteristic of cone photoreceptor cells, constitute an immortalized mouse photoreceptor cell line (by the expression of simian virus 40 T antigen) [42]. We cultured the 661W cells in Dulbecco’s modified Eagle’s media (DMEM) containing 10% phosphate buffer solution (PBS) and 1% penicillin–streptomycin (100 U/mL penicillin and 100 μg/mL streptomycin) at 37 °C and 5% CO_2_. Cells were passaged by trypsinization every 3–4 days and used for experiments at the second to fifth passage. For the experiment, the cells were exposed to either normal (5 mM/mL) or high glucose (35 mM/mL) concentration. In addition, the cells were pretreated with 10, 20, or 50 μM AST (Sigma, St. Louis, MO, USA) for 2 h prior to high glucose treatment. After 24 h treatment, the cells were collected and further analyzed. Two different inhibitors, PI3K inhibitor (LY294002, 20 μM, (Sigma, St. Louis, MO, USA)) and Nrf2 inhibitor (ML385, 10 μM, (Selleck Chemicals, Houston, TX, USA)) were used to determine the causal relationship between AST and the changes in other antioxidative molecules. The cultured cells were pretreated with either of the inhibitors and 50 μM AST simultaneously for 2 h, followed by high glucose treatment for 24 h.

### 2.2. Cell Viability Assay

Cell viability assay was performed with CyQUANT MTT Cell Viability Assay Kit (Invitrogen-Life Technologies Inc., Gaithersburg, MD, USA). The cells were seeded in 96-well plates (1 × 10^4^ cells per well) and incubated at 37 °C. Different concentrations of AST were added to the cells exposed to high glucose. After 24 h incubation, 5 mg/mL 3-(4,5-dimethylthiazol-2-yl)-2,5-diphenyltetrazolium bromide (MTT) was added to each well and incubated at 37 °C for 4 h. The culture medium supernatant was removed, and the formazan was dissolved with dimethyl sulfoxide (DMSO) for 30 min at 25 °C. The absorbance was measured at 570 nm with a microplate reader (Bio-Rad Laboratories, Inc., Hercules, CA, USA).

### 2.3. Analysis of Apoptosis by Terminal Deoxynucleotidyl Transferase dUTP Nick End Labeling (TUNEL)

We used TUNEL to detect 661W cell apoptosis. TUNEL was performed using a commercial kit (Millipore Corp., Billerica, MA, USA) according to the manufacturer’s instructions. Positive controls were cultured cells incubated with DNase I prior to the labeling procedure, and sham controls were cells stained with label solution containing no terminal transferase.

### 2.4. Detection of Intracellular ROS

We used Image-IT Green Reactive Oxygen Species Detection Kit (Invitrogen-Life Technologies Inc., Gaithersburg, MD, USA) to determine the level of ROS under different conditions. Intracellular ROS levels were measured using 2’,7’-dichlorodihydrofluorescein diacetate (2’,7’-DCFDA) oxidation. For the detection of ROS, 661W cells were exposed to 10 μM 2’,7’-DCFDA; in the presence of ROS, bright green fluorescence could be detected by a fluorescence microscope (Thermo Fisher Scientific Inc., Waltham, MA, USA).

### 2.5. Quantitative Detection of Nrf2 and ROS-Induced Cellular Oxidation Using Immunocytochemistry (ICC)

DNA oxidation, lipid peroxidation, and protein oxidation levels were determined by detecting the expression of 8-hydroxydeoxyguanosine (8-OHdG), acrolein, and nitrotyrosine, respectively, using ICC according to a previously published protocol [43]. In brief, the cultured cells were simultaneously blocked and permeabilized with 0.2% Triton in PBS containing 5% goat serum for 1 h at room temperature. Then, cells were incubated with primary antibodies diluted in blocking solution overnight at 4 °C, followed by incubation with the appropriate fluorescent secondary antibodies (all diluted 1:200) in blocking solution for 3 h at room temperature. Primary antibodies included anti-Nrf2, antiacrolein, antinitrotyrosine, and anti-8-OHdG (all from Abcam, Cambridge, MA, USA). Nuclei were counterstained with DAPI.

The quantitative protein expression measurements were done by densitometric methods as previously described [43]. The relative density of immunostaining was analyzed by an immunostaining index comparing 661W cells treated with different AST concentrations and high glucose condition versus low glucose condition (as normal reference).

### 2.6. Determination of Changes in Mitochondrial Membrane Potential

The mitochondrial membrane potential was measured using a fluorescence reader with JC-1 stain (Sigma, St. Louis, MO, USA) and a lipophilic cationic probe to detect the extent of mitochondrial dysfunction. The 661W cells were seeded in 96-well plates (1 × 10^4^ cells per well) and incubated at 37 °C. Then, different concentrations of AST were added to the cells exposed to high glucose. After 24 h incubation, 5 μL of JC-1 staining solution was added to each well, and the plate was incubated at 37 °C for 15 min. The fluorescence intensity for both J-aggregates with Texas Red (healthy cells, excitation/emission = 560/595 nm) and JC-1 monomers with FITC (apoptotic or unhealthy cells, excitation/emission = 485/535 nm) were measured with a microplate reader (Bio-Rad Laboratories, Inc., Hercules, CA, USA). For microscopy, the cells were incubated in 24-well plates. Then, 50 μL of JC-1 staining buffer was added to 1 mL of culture medium. After incubation at 37 °C for 30 min, the cells were observed using fluorescence microscopy. J-aggregates and JC-1 monomers were detected with settings designed to detect Texas Red and FITC, respectively.

### 2.7. Preparation of RNA and cDNA

Total RNA was extracted from the retinas using TRIzol reagent (Invitrogen-Life Technologies Inc., Gaithersburg, MD, USA). For each sample, 1 μg of total RNA was incubated with 300 ng of Oligo dT for 5 min at 65 °C and reverse-transcribed into cDNA using 80 U of Moloney murine leukemia virus reverse transcriptase per 50 μg reaction sample for 1 h at 37 °C. The reaction was stopped by heating the samples for 5 min at 90 °C.

### 2.8. Quantitative Analysis of mRNA Levels

We performed polymerase chain reaction (PCR) on the resultant cDNA from each sample using specific primers. The amplification was performed using a thermocycler (Applied Biosystems, Waltham, MA, USA). The 25 μL reaction mixture contained 5 μL of cDNA, 1 μL of sense and antisense primers, 200 μM of each deoxynucleotide (DTT), 5 μL of 10× Taq polymerase buffer, and 1.25 U of GoTaq polymerase (Applied Biosystems, Waltham, MA, USA). The PCR reactions were performed using an annealing temperature of 56 °C with GoTaq polymerase, cDNA, and the following primers: sense 5′-ATGACACCAAGGACCAGAGC and antisense 5′-GTAAGGACCCATCGGAGAAGC for HO-1; sense 5′-TATCCTGCCGAGTCTGTTCTG and antisense 5’-AACTGGAATATCACAAGGTCTGC for NQO1; and sense 5′-CGACTTCAACAGCAACTCCCACTC and antisense 5’-TGGGTGGTCCAGGGTTTCTTACTC for glyceraldehyde 3-phosphate dehydrogenase (GAPDH). The DNA fragments were amplified for 25–30 cycles (30 s at 94 °C; 1 min at 50–52 °C; 1 min at 72 °C), followed by a final 7 min extension step at 72 °C. The amplified products were further analyzed. Levels of each mRNA were normalized to those of GAPDH mRNA.

### 2.9. Protein Extractions and Western Blot Analysis

We extracted total proteins from the 661W cells using radioimmunoprecipitation assay (RIPA) lysis buffer (0.5 M Tris-HCl (pH 7.4), 1.5 M NaCl, 2.5% deoxycholic acid, 10% NP-40, 10 mM EDTA, and 10% protease inhibitors). Before analysis, the protein samples were separated on a 10% sodium dodecyl sulfate (SDS)–polyacrylamide gel and transferred to a polyvinylidene difluoride membrane. The membranes were probed using the following antibodies: anti-HO-1; anti-NQO1 (from Abcam, Cambridge, MA, USA); anti-PI3K; anti-Akt; anti-phospho-Akt(p-Akt); anti-caspase-3; anti-poly(ADP-ribose) polymerase (PARP) (from Cell Signaling Technology, Danvers, MA, USA); and anti-GAPDH (Millipore Corp., Billerica, MA, USA). Immunodetections were done using enhanced chemiluminescence (Pierce Biotechnology, Rockford, IL, USA) according to the manufacturer’s instructions. Protein levels were determined using densitometry analysis of the protein bands.

### 2.10. Nuclear Protein Extraction and Electrophoretic Mobility Shift Assay (EMSA)

The 661W cells treated with low glucose, high glucose, and different concentrations of AST were harvested separately. Nuclear proteins were prepared as described before [43]. In brief, the cells were trypsinized, resuspended, and homogenized in ice-cold buffer A (10 mM 4-(2-hydroxyethyl)-1-piperazineethanesulfonic acid (HEPES) (pH 7.9), 1.5 mM KCl, 10 mM MgCl2, 1.0 mM dithiothreitol (DTT), and 1.0 mM phenylmethylsulfonyl fluoride (PMSF)). The cell suspensions were homogenized and centrifuged for 10 min at 5000× *g* at 4 °C. The sediment was resuspended in 200 μL of buffer B (20 mM HEPES (pH 7.9), 25% glycerol, 1.5 mM MgCl2, 420 mM NaCl, 0.5 mM DTT, 0.2 mM EDTA, 0.5 mM PMSF, and 4 μM leupeptin). Then, the samples were incubated for 30 min on ice and centrifuged for another 30 min at 12,000× *g* at 4 °C. The supernatants containing the nuclear proteins were collected for further analysis. A bicinchoninic acid assay kit, with bovine serum albumin as the standard, was used to determine the protein concentration. EMSA was performed with a Nrf2 DNA-binding protein detection system (Abcam, Cambridge, MA, USA) according to the manufacturer’s instructions. Ten micrograms of nuclear protein was incubated with a biotin-labeled Nrf2 consensus oligonucleotide probe (5′-GCACAAAGCGCTGAGTCACGGGGAGG-3′) for 30 min in binding buffer. The specificity of the DNA/protein binding was then determined by adding a 100-fold molar excess of unlabeled Nrf2 oligonucleotide for competitive binding, 10 min before adding the biotin-labeled probe.

### 2.11. Statistical Analyses

The results are expressed as the mean ± standard deviation where applicable. The data were analyzed using SPSS software (SPSS 22.0; SPSS Inc., Chicago, IL, USA). The Mann–Whitney *U* test was used to calculate the differences between the means of different experimental groups. *p* values of less than 0.05 were considered significant.

## 3. Results

### 3.1. Astaxanthin Protects 661W Cells from Apoptosis under High Glucose Conditions

**Figure 1:**
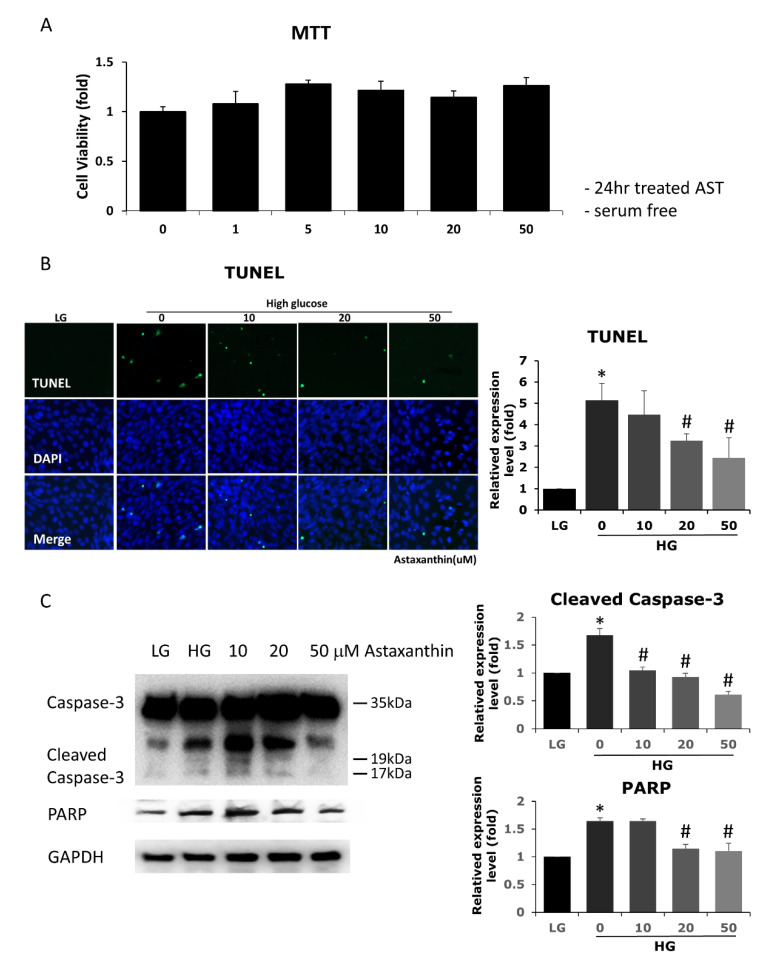
Effects of AST on cell viability and high glucose-induced apoptosis in 661W cells. (**A**) Cell viability was evaluated using MTT assay upon treatment with different concentrations of AST for 24 h. AST-treated and control cells showed similar cell viability. (**B**) Increased cell apoptosis was noted under high glucose environment, demonstrated by TUNEL assay and protein expression (western blots, **C**). AST reduced apoptosis at 20 and 50 μM concentration. (**C**) Decreased cleaved caspase-3 and PARP protein expression seen in western blots indicated that apoptosis was attenuated by AST. AST = astaxanthin; HG = high glucose; LG = low glucose; MTT = 3-(4,5-dimethylthiazol-2-yl)-2,5-diphenyltetrazolium bromide; TUNEL = terminal deoxynucleotidyl transferase dUTP nick end labeling; PARP = poly(ADP-ribose) polymerase; GAPDH = glyceraldehyde 3-phosphate dehydrogenase. * Significantly different from control group (*p* < 0.05); # significantly different from high glucose-treated group without AST (*p* < 0.05). The Mann–Whitney *U* test was used to calculate the differences between the means of different experimental groups (three repeats per experiment, three wells per repeat, five images per well analyzed).

To evaluate the potential role of AST as a therapeutic agent, we first tested the safety of different concentrations (1, 5, 10, 20, and 50 μM) of AST on 661W cells. The cell viability was not affected by treatment with up to 50 μM AST, as shown by MTT assay (Figure 1A). Therefore, 10, 20, and 50 μM AST were used for the following experiments. To evaluate the protective effect of AST in 661W cells under high glucose conditions, we used the TUNEL assay to detect apoptosis. The results demonstrated a dose-dependent protective effect of AST against 661W cell apoptosis in a high glucose environment (Figure 1B), with a significantly decreased number of apoptotic cells upon treatment with 20 and 50 μM AST. The antiapoptotic effect of AST was further confirmed by decreased caspase-3 cleavage and decreased PARP in AST-treated 661W cells (Figure 1C).

### 3.2. Astaxanthin Reduces ROS and ROS-Related Mitochondrial Damage

**Figure 2:**
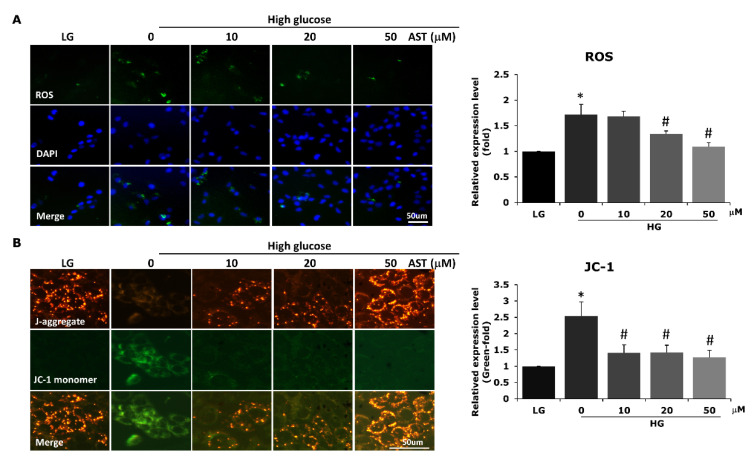
AST reduced ROS and ROS-related mitochondrial damage. (**A**) 661W cells pretreated with different concentrations of AST for 2 h were cultured under high glucose environment. Intracellular ROS levels were detected by measuring the green fluorescence of 2′,7′-dichlorodihydrofluorescein diacetate oxidation. ROS levels were significantly reduced in cells pretreated with 20 and 50 μM of AST. (**B**) Mitochondrial membrane potential was evaluated by JC-1 staining. The results showed decreased mitochondrial damage after AST treatment. AST = astaxanthin; HG = high glucose; LG = low glucose; ROS = reactive oxygen species. * Significantly different from control group (*p* < 0.05); # significantly different from high glucose-treated group without AST (*p* < 0.05). The Mann–Whitney *U* test was used to calculate the differences between the means of different experimental groups (three repeats per experiment, three wells per repeat, five images per well analyzed).

To understand if the observed attenuation in apoptosis resulted from the antioxidative effect of AST, we measured the intracellular ROS levels in cells treated with different concentrations of this compound. AST reduced the levels of ROS increased by high glucose treatment in 661W cells at 20 μM and 50 μM concentration (Figure 2A). The ROS-related mitochondrial damages, detected by JC-1 staining, were also reduced by AST treatment (Figure 2B).

### 3.3. Astaxanthin Reduces ROS-Related Lipid, Protein, and DNA Damage

**Figure 3:**
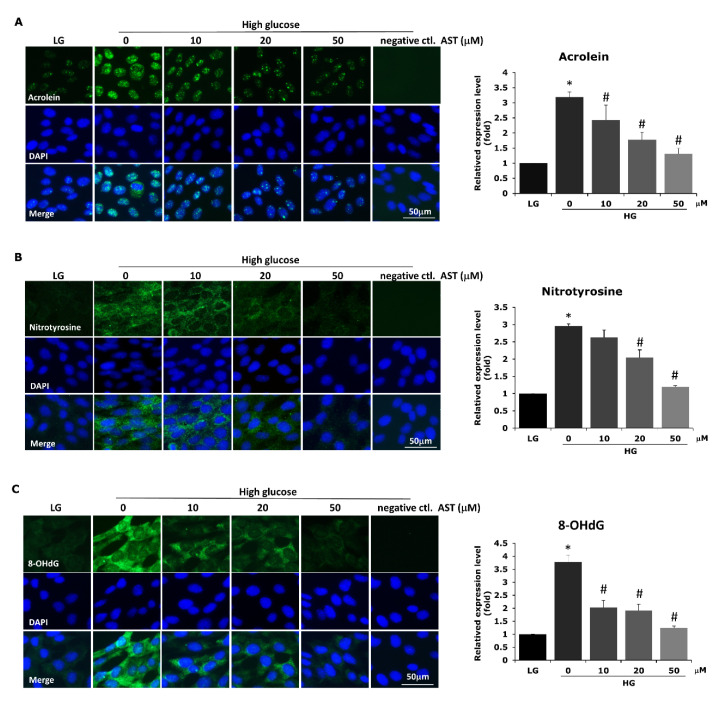
AST reduces ROS-related lipid, protein, and DNA damage in high glucose-treated 661W cells. To confirm that high glucose-induced ROS-related damage was also attenuated by AST treatment, we measured the expression of acrolein (**A**), nitrotyrosine (**B**), and 8-OHdG (**C**) by immunocytochemistry to indicate the oxidative damages to lipids, proteins, and DNA, respectively. Significantly decreased oxidative damage was noted under AST treatment. AST = astaxanthin; HG = high glucose; LG = low glucose; ROS = reactive oxygen species. * Significantly different from control group (*p* < 0.05); # significantly different from high glucose-treated group without AST (*p* < 0.05). The Mann–Whitney *U* test was used to calculate the differences between the means of different experimental groups (three repeats per experiment, three wells per repeat, five images per well analyzed).

Increased intracellular ROS levels can cause further damage to lipids, proteins, and DNA, which may eventually lead to apoptosis. We therefore evaluated the expression of acrolein, nitrotyrosine, and 8-OHdG using different concentrations of AST. The levels of all three surrogate markers decreased with increasing concentrations of AST, indicating the antioxidative capacity of AST treatment (Figure 3). Significantly reduced damage to lipids and DNA (indicated by acrolein and 8-OHdG, respectively) and to proteins (indicated by nitrotyrosine) was observed with at least 10 or 20 μM AST, respectively.

### 3.4. Astaxanthin Reduces ROS through Upregulation of Phase II Enzymes

**Figure 4:**
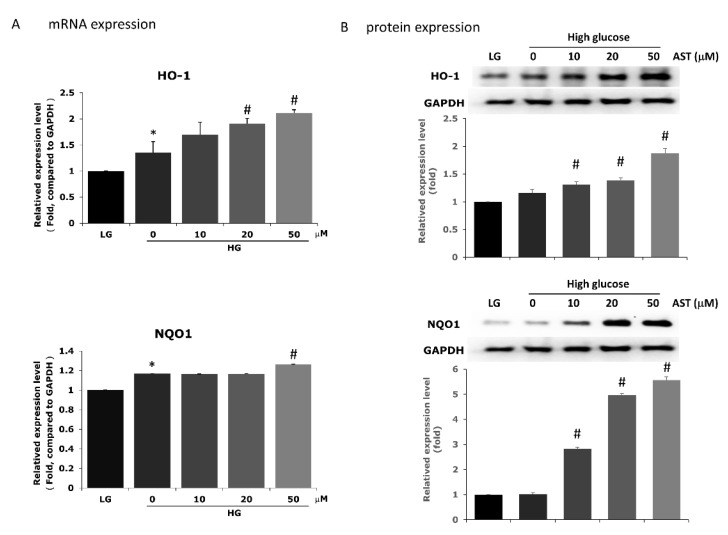
AST promotes the expression of Phase II enzymes HO-1 and NQO1 in 661W cells. The expression of HO-1 and NQO1 was determined in AST-treated 661W cells by measuring the mRNA levels with qPCR (**A**) and the corresponding protein levels by western blot (**B**). The measurements were normalized to GAPDH. AST = astaxanthin; HO-1 = Heme oxygenase-1; HG = high glucose; LG = low glucose; NQO1 = NAD(P)H dehydrogenase. * Significantly different from control group (*p* < 0.05); # significantly different from high glucose-treated group without AST (*p* < 0.05). The Mann–Whitney *U* test was used to calculate the differences between the means of different experimental groups.

The Phase II enzymes HO-1 and NQO1, which exhibit antioxidative properties and are known to reduce intracellular ROS, have been reported to be upregulated by AST [36,38,44]. To confirm if the reduction in ROS induced by AST in our study was associated with a change in Phase II enzyme expression, we evaluated both the mRNA and protein levels of HO-1 and NQO1. We found that Phase II enzymes were upregulated both at the mRNA and protein levels after AST treatment in a dose-dependent fashion (Figure 4).

### 3.5. Astaxanthin Activates PI3K/Akt Pathway and Upregulates the Expression of Nrf2

**Figure 5:**
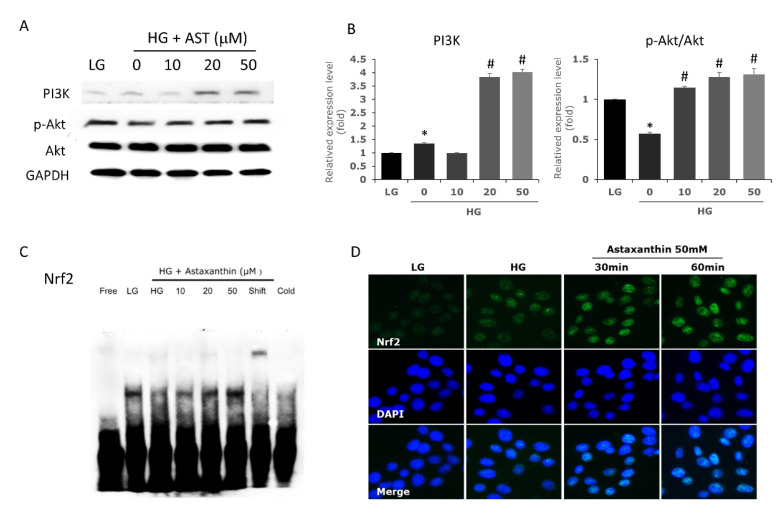
AST upregulates the expression of the PI3K/Akt/Nrf2 pathway in 661W cells. (**A**,**B**) The expression of PI3K and phosphorylated Akt proteins were detected by western blot. 661W cells were pretreated with different concentrations of AST for 2 h and grown in high glucose for 24 h. (**C**) Nrf2 levels in nuclear protein extracts were determined by electrophoretic mobility shift assay. (**D**) Immunocytochemistry confirmed the increased expression of Nrf2 after AST treatment. AST = astaxanthin; HG = high glucose; LG = low glucose. * Significantly different from control group (*p* < 0.05); # significantly different from high glucose-treated group without AST (*p* < 0.05). The Mann–Whitney *U* test was used to calculate the differences between the means of different experimental groups.

We further evaluated the activation of the PI3K/Akt pathway and the expression of Nrf2 under AST treatment to better understand how AST protects 661W cells from high glucose-induced damage. Treatment of 661W cells cultured in high glucose with 20 and 50 μM AST increased PI3K protein levels, which further increased the downstream p-Akt/Akt ratio (Figure 5A,B). In the same culture conditions, we found increased nuclear expression of Nrf2 upon treatment with AST (Figure 5C). Moreover, activation of Nrf2 was observed 30 min after treatment with 50 μM AST (Figure 5D). These results indicate that AST activates the PI3K/Akt/Nrf2 pathway, which further contributes to decreasing the ROS generated in a high glucose environment.

### 3.6. Inhibition of Both PI3K and Nrf2 Attenuate the Antioxidative Effect of AST

**Figure 6:**
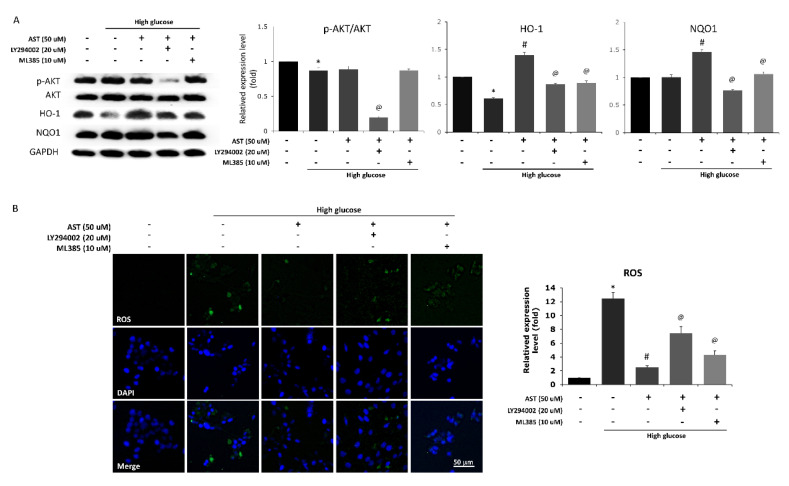
Blocking the PI3K/Akt/Nrf2 pathway abolishes the antioxidative effect of AST in 661W cells. Cells were administered PI3K inhibitor (LY294002, 20 μM) and Nrf2 inhibitor (ML385, 10 μM) along with AST. (**A**) The protein expression of Akt, p-Akt, HO-1, and NQO1 was detected by western blot. The PI3K inhibitor resulted in lower p-Akt/Akt ratio, HO-1, and NQO1 expression compared with AST treatment alone. Meanwhile, Nrf2 inhibition resulted in decreased expression of HO-1 and NQO1 but not the p-Akt/Akt ratio. (**B**) Both inhibitors counteracted the protective effect of AST and significantly increased the ROS levels, AST = astaxanthin; ROS = reactive oxygen species. * Significantly different from control group (*p* < 0.05); # significantly different from high glucose-treated group without AST (*p* < 0.05); @ significantly different from high glucose-treated group with 50 μM AST. The Mann–Whitney *U* test was used to calculate the differences between the means of different experimental groups (three repeats per experiment, three wells per repeat, five images per well analyzed).

To confirm whether the protective effects of AST act through the PI3K/Akt/Nrf2 pathway, we added the PI3K inhibitor LY294002 (20 μM) and Nrf2 inhibitor ML385 (10 μM) and observed the corresponding changes in Phase II enzyme production and ROS levels. In the presence of PI3K inhibitor, downstream p-Akt decreased accordingly. Moreover, the levels of both HO-1 and NQO1 were lower upon treatment with the PI3K inhibitor than with AST alone (Figure 6A). The Nrf2 inhibitor also downregulated the AST-enhanced expression of HO-1 and NQO1 while not affecting p-Akt. Both PI3K and Nrf2 inhibitors attenuated the reduction in ROS induced by AST (Figure 6B). These results confirmed the sequential changes and causal relationship between PI3K/Akt pathway, Nrf2 and Phase II enzyme expression, and apoptosis with the protective effects of AST in photoreceptor cells.

## 4. Discussion

Astaxanthin possesses unique chemical properties derived from its distinctive molecular structure, including two hydroxyl groups, two carbonyl groups, and 11 conjugated ethylenic double bonds. In contrast to other carotenoids, AST can be esterified, has a more polar nature, and displays greater antioxidant capacity, which could be explained by the presence of the hydroxyl and keto moieties on each ionone ring [19]. AST was recently reported to significantly suppress oxidative stress and protect various cells from apoptosis [30,32,33,41,45]. In this study, we demonstrated that AST could protect photoreceptor cells from oxidative stress through activation of the PI3K/Akt/Nrf2 signaling pathway in high glucose environments, which further reduced apoptosis and could potentially prevent the progression of DR.

The correlation between oxidative stress and the development of DR has been previously demonstrated [7,8,9,10,11,12]. Increased oxidative stress and production of ROS cause tissue injury through peroxidation of lipids, carbohydrates, proteins, and DNA [46], with the concomitant production of oxidative biomarkers, such as acrolein, carbonylated proteins, nitrotyrosine, and 8-OHdG [47,48]. In this study, we confirmed the harmful effects of high glucose by showing an increase in ROS, oxidative biomarkers 8-OHdG, nitrotyrosine, and acrolein, as well as apoptosis. In addition, our results also demonstrated that the oxidative stress and consequent ROS-induced damage to DNA, lipids, and proteins could be ameliorated by the antioxidative effect of AST. The overall reduction in oxidative stress mediated by AST further led to decreased apoptosis of photoreceptor cells. Baccouche et al. [49] found that short-term use of AST could reduce retinal damage in fat sand rats. In their study, while the serum glucose levels were similar in the AST-treated animals and the control group, the cellular damage in Muller cells and retinal ganglion cells was reduced by AST treatment through increased HO-1 production. Dong et al. [50] also reported a protective effect of AST against the oxidative stress induced by diabetes in retinal ganglion cells. Another study using streptozotocin-induced diabetic rats demonstrated the antioxidative effect of AST in the retina in a high glucose environment [51]. In this study, after AST treatment, the retinal thickness was preserved not only in the inner but also in the outer retinal layers. The cellular damage in DR is not limited to retinal ganglion cells. Photoreceptor loss in DR due to increased apoptosis has also been reported [52]. Our results confirm that the antiapoptotic effect of AST is not limited to ganglion cells and Muller cells but also extends to photoreceptors, indicating the potential universal benefits of AST treatment in DR.

The Nrf2–ARE pathway plays an important role in cellular resistance to oxidative stress [34,35,44]. Nrf2 is a transcription factor that binds to the ARE and promotes the expression of Phase II enzymes [36]. In the absence of oxidative damage, Nrf2 interacts with the chaperone Keap1; conversely, in an oxidant environment, Nrf2 dissociates from Keap1 and translocates to the nucleus in its activated form, where it binds to the ARE and induces the expression of Phase II enzymes, such as HO-1 and NQO1 [37]. Several studies have demonstrated the beneficial effects of Nrf2 activation in DR. Fenofibrate was reported to activate Nrf2, increase HO-1 and NQO1 expression, and reduce oxidative damage in diabetic mice [53]. Sulforaphane also exhibited similar antioxidative effects in streptozotocin-induced diabetic rats and high glucose-treated Muller cells through the activation of the Nrf2 pathway [54]. Furthermore, activation of Nrf2, either through DJ-1 overexpression [55] or through Nrf2 activators [56], could provide additional benefits to improve retinal pericyte survival and reduce vascular endothelial growth factor-induced cell migration in human retinal microvascular endothelial cells, both involved in the development of DR. In the current study, we have further demonstrated that the Nrf2–ARE pathway can be activated by AST, thereby increasing the expression of HO-1 and NQO1, which in turn attenuates ROS-mediated and intracellular oxidative damage and further protects photoreceptor cells from high glucose-induced apoptosis.

AST can ameliorate cell apoptosis via more than one pathway, including ERK, NF-κB, and PI3K/Akt [27,57,58,59]. Among these, the PI3K/Akt pathway is a prominent regulator of numerous proteins involved in cell survival through profound antioxidative and antiapoptotic actions [60]. Studying apoptosis after burn in an animal model, Guo et al. demonstrated a dose-dependent effect of AST in increasing p-Akt and decreasing cleaved caspase-3 levels [61]. In diabetic rats, the cognitive functions could also be preserved by AST through activation of the PI3K/Akt pathway and downregulation of caspase-3 expression [62]. In RPE cells, Li et al. [41] reported that oxidative stress was attenuated by AST. They further demonstrated that AST upregulates Nrf2 and Nrf2-related Phase II enzymes through activation of the PI3K/Akt pathway. Collectively, these pieces of evidence support the involvement of the PI3K/Akt pathway in the antiapoptotic effect of AST. Nevertheless, the cellular response to AST may differ among distinct cell types and with diverse environmental stimuli. In a hamster model of oral cancer, Kavitha et al. [63] found downregulated PI3K and p-Akt in AST-treated animals, which further led to caspase-induced apoptosis. Therefore, we specifically focused here on the response of photoreceptor cells. Our study clearly demonstrated that the PI3K/Akt pathway was upregulated in the presence of AST and that downstream Nrf2 expression was enhanced along with an increased expression of HO-1 and NQO1, which eventually reduced caspase activity and ameliorated apoptosis in high glucose-treated photoreceptor cells. Both PI3K inhibitor (LY294002) and Nrf2 inhibitor (ML385) counteracted the protective effects of AST and attenuated the expression of HO-1 and NQO1, indicating the importance of PI3K/Akt/Nrf2 signaling in AST-mediated cellular protection.

Systemic AST supplementation could function in the eye and potentially protect the retina from various diseases by reducing oxidative stress. Oral AST supplementation has been used in previous randomized controlled trials and was found to suppress the aqueous vascular endothelial growth factor levels and peroxide production in humans [64,65]. An earlier trial using AST in combination with lutein and other antioxidants also reported improved central visual function in patients with age-related macular degeneration [66]. The results from our study, along with those from previous studies, imply the potential benefits of AST in DR. Taken together with the benefits of AST in increasing serum insulin and glucose metabolism [24,67,68], the role of oral AST supplementation in the prevention and treatment of DR warrants further evaluation in clinical trials.

There were a few limitations to our study. First, we treated the photoreceptor cells with AST before the high glucose administration; therefore, we evaluated only the effects of AST on the acute response of cells to high glucose but not on the cells that had already developed a certain degree of damage under the high glucose environment. Previous in vivo studies have demonstrated that AST administered after the induction of diabetes in different animal models may reduce the levels of oxidative stress mediators and preserve retinal function [49,50,51]. These results indicate that AST may potentially be used for the treatment of high glucose-induced damage, but further studies are needed to confirm the cellular responses of AST administered after the occurrence of cellular damage. Second, we only used a fluorescent probe to detect ROS. Other methods, including spectrophotometry, chromatography, and electron spin resonance, have also been proposed to increase the sensitivity and specificity of ROS detection [69]. Nevertheless, we used other ancillary tests to confirm our detection of ROS, and the results of JC-1 staining and the detection of oxidative stress mediators support our conclusions.

## 5. Conclusions

The data presented in this study indicate that AST could protect photoreceptor cells from apoptosis secondary to high glucose-induced oxidative stress. In addition, we have demonstrated that the protective effects of AST are mediated by upregulated Nrf2 and that increased expression of downstream Phase II enzymes results from activation of the PI3K/Akt pathway. Thus, AST should be considered as a nutritional supplement that could benefit patients with diabetes, especially in view of preventing the visual loss in DR.
